# Allied health – 3003. The relation of cachexia to the risk of hospitalization with fixed airway obstruction

**DOI:** 10.1186/1939-4551-6-S1-P180

**Published:** 2013-04-23

**Authors:** June-Hyuk Lee, An-Soo Jang, Sung-Woo Park, Dojin Kim, June-Hyuk Lee

**Affiliations:** 1 Respiratory and Allergy, Soonchunhyang University Bucheon Hospital, Bucheon, South Korea; 2 Soonchunhyang University Bucheon Hospital, South Korea

## Background

Body mass index (BMI) have suggested to be related with obstructive lung diseas. BMI was associated with the extent of emphysema distribution, but has still not been known about the association to degree of airway obstruction. Wetried to determine whether cachexia or BMI is risk factor of hospitalization in asthma with fixed airway obstruction.

## Methods

245 asthmatic patients were enrolled. Cachexia was defined as BMI < 18. The severity of asthma was classified into grade based on GOLD criteria.

## Results

BMI was significantly associated with the severity of airway obstruction. BMI was significantly associated with lung function. The severity of airway obstruction was significantly associated with the frequency and duration of hospitalization (*P* = 0.02, *P* = 0.007 respectively). The cachexia was not associated with the frequency of hospitalization (*P* = 0.21), while it was significantly associated with the duration of hospitalization (*P* = 0.019).

## Conclusions

Cachexia can be a risk factor for the longer duration of hospitalization being independent of degree of fixed airway obstruction.

